# Broadband variable beamsplitter made of a subwavelength-thick metamaterial

**DOI:** 10.1515/nanoph-2025-0512

**Published:** 2025-12-09

**Authors:** Yasuhiro Tamayama, Yugo Shibata

**Affiliations:** Department of Electrical, Electronics and Information Engineering, 52756 Nagaoka University of Technology, 1603-1 Kamitomioka, Nagaoka, Niigata 940-2188, Japan

**Keywords:** metamaterial, metasurface, Brewster effect, beamsplitter, radiation pattern, temporal coupled-mode theory

## Abstract

We propose and validate a method for designing a broadband variable beamsplitter using a metamaterial with subwavelength thickness. Through theoretical analysis and numerical simulations, we demonstrate that the reflectance-to-transmittance ratio of a single-layer resonant metamaterial at its resonance frequency can be controlled by varying the spatial arrangement of the constituent meta-atoms, without altering their individual structures. Building on this theory, we further conjecture a method for achieving a frequency-independent reflectance-to-transmittance ratio across a broad spectral range. Numerical results confirm that a metamaterial with subwavelength thickness can be engineered to function as a broadband variable beamsplitter using the proposed approach. These findings contribute to the advancement of techniques for splitting and combining electromagnetic waves in compact systems.

## 1 Introduction

There has been considerable interest in controlling electromagnetic waves using metasurfaces, which serve as the two-dimensional counterparts of metamaterials. Researchers have developed compact optical components for a wide range of applications, including wavefront control [1], [2], [3], [4], [5], [6], [7], [8], [9], [10], dynamic manipulation of wave propagation [11], [12], [13], [14], [15], [16], [17], [18], [19], [20], [21], and efficient generation of nonlinear phenomena [22], [23], [24], [25], [26], [27], [28], [29], [30], [31].

While metasurfaces are essential for miniaturization of optical systems, it is important to note that not all functionalities can be realized using one single-layer planar metasurface, which supports only electric dipole responses. For instance, arbitrary complex reflectance or transmittance cannot generally be achieved with such structures [2], [32], [33]. To enable arbitrary control of complex reflectance, a ground plane must be added [34], [35], [36], whereas arbitrary control of complex transmittance requires the design of a Huygens metasurface [2], [3], [5], [8], [37], [38], [39], [40], [41], [42], [43], [44], [45], [46]. Huygens metasurfaces typically consist of thick and/or multilayer structures and exhibit not only electric dipole responses but also magnetic dipole and high-order multipole responses. Another approach to overcoming the limitation on the properties of single-layer planar metasurfaces involves the steric arrangement of meta-atoms [47], [48].

As outlined above, arbitrary complex reflectance or transmittance can be achieved by stacking multiple metasurfaces, increasing their thickness, and/or sterically arranging the constituent meta-atoms. While previous studies have extensively explored the control of either complex reflectance or complex transmittance, the control of the ratio between reflectance and transmittance has received little attention. Beamsplitters are essential components in various applications, including interferometry, spectroscopy, and imaging systems. Therefore, developing techniques to control the reflectance-to-transmittance ratio using metasurfaces would be highly beneficial for advancing electromagnetic wave technologies.

To address this, we focus on the distinction between single-layer planar metasurfaces and Brewster metafilms developed in our previous studies [47], [49], [50]. Brewster metafilms are single-layer metamaterials composed of meta-atoms arranged such that the oscillation direction of the induced electric dipoles aligns with the propagation direction of the reflected wave. For planar metasurfaces, the incident wave is completely reflected at the resonance frequency [51]. In contrast, for Brewster metafilms, the incident wave is fully transmitted regardless of frequency. Although these two types of metamaterials exhibit markedly different reflectance and transmittance characteristics, the only difference in their microscopic responses lies in the oscillation direction of the induced electric dipoles.

This observation suggests that the optical properties of single-layer metamaterials can be tuned continuously from perfect reflection to perfect transmission solely by modifying the arrangement of the constituent meta-atoms. In this study, we theoretically and numerically demonstrate that the reflectance-to-transmittance ratio at the resonance frequency in single-layer resonant metamaterials can be arbitrarily controlled by adjusting the spatial configuration of meta-atoms. Furthermore, we propose a method for achieving a frequency-independent reflectance-to-transmittance ratio over a broad frequency range and numerically verify that a broadband variable beamsplitter can be designed using this approach.

## 2 Theory

**Figure 1:**
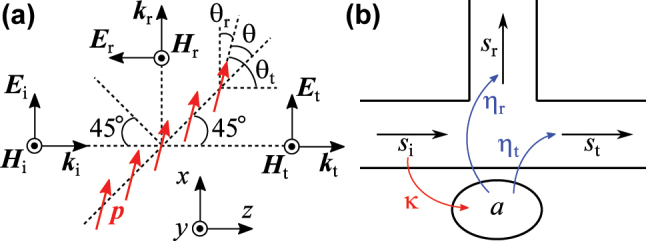
Theoretical model of the proposed variable beamsplitter. (a) Relationship between the oscillation direction of the electric dipole *p* induced in a single-layer metamaterial and the incident, reflected, and transmitted electromagnetic fields. (b) Schematic of a resonator system coupled to three propagation modes. The subscripts of “i,” “r,” and “t” indicate the incident, reflected, and transmitted waves, respectively.

To construct a theoretical framework for controlling the ratio of the reflectance to the transmittance, we begin by analyzing the radiation from a single-layer metamaterial illuminated by a plane electromagnetic wave, as illustrated in Figure 1(a). The constituent meta-atoms are arranged periodically along the 45° direction relative to the *z*-axis and along the *y*-axis. Each meta-atom supports only an electric dipole, with its oscillation direction oriented at 45° + *θ* with respect to the *z*-axis. An *x*-polarized plane electromagnetic wave is incident from the −*z* direction at an angle of 45°. In this configuration, the metamaterial can radiate electromagnetic waves along both the *x*- and *z*-directions.

For simplicity, we model the induced electric dipole infinitesimal. The radiation pattern of such a dipole is proportional to sin *ϕ*, where *ϕ* is the angle between the dipole’s oscillation direction and the propagation direction of the radiated wave [52]. Therefore, the ratio of the amplitude of the radiation in the *x*-direction to that in the *z*-direction is given by [53]:

(1) $$k=\frac{\sin\theta_{\text{r}}}{\sin\theta_{\text{t}}}=\frac{\sin\left(45{}^{\circ}-\theta \right)}{\sin\left(45{}^{\circ}+\theta \right)}=\frac{1}{\tan\left(45{}^{\circ}+\theta \right)},$$

where *θ*_r_ and *θ*_t_ are the angles between the oscillation direction of the induced electric dipoles and the propagation directions of the reflected and transmitted waves, respectively. When *θ* = 0°, *k* = 1, indicating equal amplitudes of radiation in the reflected and transmitted directions. This corresponds to single-layer planar metasurfaces, where electric dipoles oscillate within the metasurface plane. As *θ* increases, *k* decreases. At *θ* = 45°, *k* = 0, meaning no radiation occurs in the reflected direction – a characteristic of Brewster metafilms. Thus, by varying *θ* from 0° to 45°, *k* can be tuned continuously from 1 to 0.

Next, we analyze the reflectance and the transmittance of a single-layer resonant metamaterial using the model shown in Figure 1(b). In this model, a resonance mode is excited by an incident wave and radiates into both the reflected and transmitted directions. It is assumed that diffraction does not occur. According to temporal coupled-mode theory [54], [55], [56], the system dynamics are governed by:

(2) $$\frac{da}{d\tau }=\left(-i\omega_{0}-\gamma_{\text{r}}-\gamma_{\text{nr}}\right)a+\kappa s_{\text{i}},$$

(3) $$s_{\text{r}}=\eta_{\text{r}}a,$$

(4) $$s_{\text{t}}=s_{\text{i}}+\eta_{\text{t}}a,$$

where *s*_i_, *s*_r_, and *s*_t_ denote the amplitudes corresponding, respectively, to the incident, reflected, and transmitted waves; *a* denotes the amplitude of the resonance mode; *κ* the coupling between the incident wave and the resonance mode; *ω*_0_ the resonance angular frequency; *η*_r_ and *η*_t_ denote the couplings to the reflected and transmitted waves; *γ*_r_ and *γ*_nr_ the radiative and nonradiative losses of the resonance mode; and *τ* denotes time. From the previous definition, the ratio *k* is expressed as:

(5) $$k=\frac{\eta_{\text{r}}}{\eta_{\text{t}}}.$$

Before calculating reflectance and transmittance, we derive the relationship between *γ*_r_ and the coupling coefficients *η*_{r,t}_. Assuming no incident wave (*s*_i_ = 0) and no nonradiative loss (*γ*_nr_ = 0), Eq. (2) yields *a* = *A* exp[(−i*ω*_0_ − *γ*_r_)*τ*], where *A* is a constant. Since all the energy is radiated into the reflected and transmitted waves, energy conservation requires $d|a{|}^{2}/d\tau =-\left(|s_{\text{r}}{|}^{2}+|s_{\text{t}}{|}^{2}\right)$ [54], [55], [56]. Substituting Eqs. (3) and (4) into this equation, we obtain:

(6) $$2\gamma_{\text{r}}=|\eta_{\text{r}}{|}^{2}+|\eta_{\text{t}}{|}^{2}.$$

Now, for a monochromatic incident wave with angular frequency of *ω*, Eq. (2) simplifies to *a* = {*κ*/[−i(*ω* − *ω*_0_) + *γ*_r_ + *γ*_nr_]}*s*_i_. Substituting into Eqs. (3) and (4), the complex reflectance *r* and the complex transmittance *t* are:

(7) $$r=\frac{s_{\text{r}}}{s_{\text{i}}}=\frac{\eta_{\text{r}}\kappa }{-i\left(\omega -\omega_{0}\right)+\gamma_{\text{r}}+\gamma_{\text{nr}}},$$

(8) $$t=\frac{s_{\text{t}}}{s_{\text{i}}}=1+\frac{\eta_{\text{t}}\kappa }{-i\left(\omega -\omega_{0}\right)+\gamma_{\text{r}}+\gamma_{\text{nr}}}.$$

Assuming *γ*_nr_ = 0, energy conservation requires |*r*|^2^ + |*t*|^2^ = 1, which leads to:

(9) −2(ω−ω0)Im(ηtκ)+2γrRe(ηtκ)+|ηr|2+|ηt|2|κ|2=0.

To satisfy this equation for all *ω*, Im(*η*_t_ *κ*) = 0 must hold, implying *η*_t_ *κ* is real. Thus, Eq. (9) reduces to:

(10) $$\eta_{\text{t}}=-\kappa^{*},$$

where Eq. (6) is used. Substituting Eqs. (5) and (10) into Eq. (6), we obtain:

(11) $$|\kappa {|}^{2}=\frac{2\gamma_{\text{r}}}{k^{2}+1}.$$

Finally, using Eqs. (5), (10), and (11), the reflectance and transmittance become:

(12) $$r=-\frac{2k}{k^{2}+1}\frac{\gamma_{\text{r}}}{-i\left(\omega -\omega_{0}\right)+\gamma_{\text{r}}+\gamma_{\text{nr}}},$$

(13) $$t=1-\frac{2}{k^{2}+1}\frac{\gamma_{\text{r}}}{-i\left(\omega -\omega_{0}\right)+\gamma_{\text{r}}+\gamma_{\text{nr}}}.$$

Based on Eqs. (12) and (13), we develop a method for controlling the ratio of the reflectance to the transmittance at the resonance frequency. At resonance *ω* = *ω*_0_, the reflectance and the transmittance simplify to:

(14) $$r\left(\omega_{0}\right)=-\frac{2k}{\left(k^{2}+1\right)\left[1+\left(\gamma_{\text{nr}}/\gamma_{\text{r}}\right)\right]},$$

(15) $$t\left(\omega_{0}\right)=\frac{k^{2}-1}{\left(k^{2}+1\right)\left[1+\left(\gamma_{\text{nr}}/\gamma_{\text{r}}\right)\right]}.$$

To examine the dependence on *k*, we set *γ*_nr_ = 0. When *k* = 1, *r*(*ω*_0_) = −1 and *t*(*ω*_0_) = 0, indicating total reflection. As *k* decreases, |*r*(*ω*_0_)| decreases and |*t*(*ω*_0_)| increases. At *k* = 0, *r*(*ω*_0_) = 0 and *t*(*ω*_0_) = −1 indicating total transmission. From Eq. (1), *k* varies from 1 to 0 as *θ* varies from 0° to 45°. Therefore, the ratio |*r*(*ω*_0_)|/|*t*(*ω*_0_)| can be arbitrarily controlled by tuning *θ* within this range.

Building on the above theory for controlling the reflectance-to-transmittance ratio at the resonance frequency, we propose a method for achieving a frequency-independent ratio across a broad frequency range. From the theoretical results, we infer that if a single-layer resonant metamaterial exhibits perfect reflection (perfect transmission) over a wide frequency band when *θ* = 0° (*θ* = 45°), then the reflectance within this band may be governed primarily by *θ* and not by frequency. Assuming the nonradiative loss of the metamaterial is negligible, the transmittance should likewise remain frequency-independent due to energy conservation. This suggests that broadband beamsplitters with tunable splitting ratios controlled by *θ* are feasible using a single-layer metamaterial that supports perfect reflection for *θ* = 0° and perfect transmission for *θ* = 45° over a broad spectral range.

## 3 Numerical simulation

**Figure 2:**
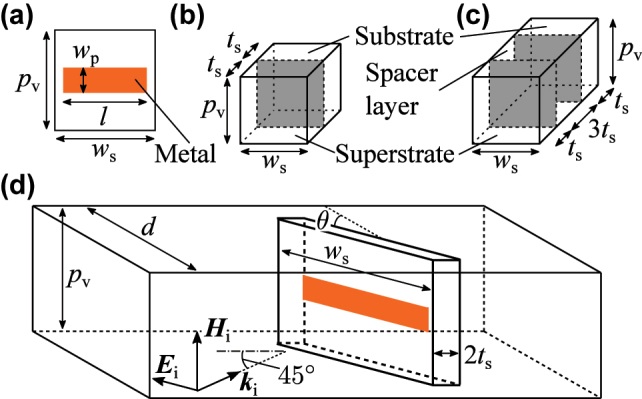
Geometry of the metamaterials and the simulation system. (a) Schematic of the structure of the dipole resonator. (b) A constituent element of the single-layer metamaterial. (c) A constituent element of the two-layer metamaterial. The dipole resonator is in the gray planes. (d) Simulation system for analyzing the reflectance and the transmittance of the metamaterials. This figure relates to the analysis of a single-layer metamaterial. The geometrical parameters used in the numerical simulation are as follows: *w*_p_ = 0.5 mm, *l* = 14.0 mm, *w*_s_ = 15.0 mm, *t*_s_ = 0.8 mm, *p*_v_ = 4.0 mm, and *d* = 16.0 mm. The relative permittivity of the substrate, superstrate, and spacer layer is taken to be 4.5(1 + i0.03).

To verify the theoretical framework for controlling the reflectance-to-transmittance ratio at the resonance frequency, we numerically analyzed the dependence of reflection and transmission spectra on *θ* for a single-layer metamaterial. Any resonant structure exhibiting only an electric dipole response can serve as the constituent meta-atom; in this study, we employed a commonly used dipole resonator, as shown in Figure 2(a). When a horizontally polarized electromagnetic wave is incident on this structure, a horizontally oscillating electric dipole is induced. The dipole resonator has a relatively high radiative loss, which is advantageous in decreasing *γ*_nr_/*γ*_r_. The dipole resonator was assumed to be fabricated on a dielectric substrate and covered with the same dielectric material to realistically lower the resonance frequency. This structure can be fabricated using standard printed circuit board technology in the microwave region.

We performed numerical simulations of the reflection and transmission spectra for the single-layer metamaterial composed of the structure shown in Figure 2(b), using COMSOL Multiphysics. The simulation setup is illustrated in Figure 2(d). The dipole resonator was modeled as a perfect electric conductor with negligible thickness. Both the substrate and superstrate were assumed to be FR-4, with a relative permittivity of 4.5(1 + i0.03). The dielectric loss of FR-4 is relatively high compared with that of the other low-loss printed circuit board substrates. For example, the dielectric loss tangent of polyphenylene ether is 0.005 and that of Rogers RT/duroid 5880 is 0.0009. This implies that we examine the influence of material loss on the reflection and transmission spectra by assuming that the substrate and superstrate have a relatively high dielectric loss. Periodic boundary conditions were applied along the horizontal boundaries to simulate periodic arrangement of the meta-atoms, as depicted in Figure 1(a). Perfectly matched layer boundary conditions were applied perpendicular to the horizontal direction. A p-polarized electromagnetic wave was incident at the incident angle of 45°, and reflection and transmission spectra were calculated for various values of *θ*.

**Figure 3:**
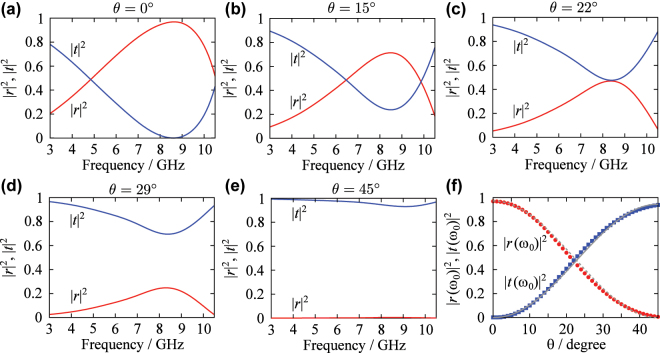
Numerically calculated reflection and transmission spectra of the single-layer metamaterial for (a) *θ* = 0°, (b) 15°, (c) 22°, (d) 29°, and (e) 45°. (f) The *θ* dependences of reflectance and transmittance at the resonance frequency. The gray solid and dashed curves are theoretical fits. The best fit of the numerically calculated data to Eqs. (14) and (15) is for *γ*_nr_/*γ*_r_ = 1.42 × 10^−2^.

Figure 3(a)–(e) shows the numerically computed reflection and transmission spectra for *θ* = 0°, 15°, 22°, 29°, and 45°. For *θ* = 0°, the reflectance |*r*|^2^ nears unity and the transmittance |*t*|^2^ nears zero at the resonance frequency 8.64 GHz (defined here as the frequency at which the magnitude of the reflectance is maximized). As *θ* increases, the magnitude of the reflectance at the resonance frequency decreases while the transmittance increases. For *θ* = 45°, |*r*|^2^ approaches zero and |*t*|^2^ approaches unity, nearly independent of frequency. Figure 3(f) shows the dependence of |*r*|^2^ and |*t*|^2^ on *θ* at the resonance frequency, along with theoretical fits based on Eqs. (14) and (15). The numerical results show excellent agreements with the theoretical predictions. The best-fit value of the parameter *γ*_nr_/*γ*_r_ is 1.42 × 10^−2^. It is confirmed from these results that the reflectance-to-transmittance ratio at resonance can be controlled by varying *θ* in a single-layer metamaterial and that *γ*_nr_/*γ*_r_ can be decreased by using the dipole resonator as the constituent element even for the case where FR-4 is used as the substrate and superstrate.

We next verify the conjectured method for achieving a frequency-independent reflectance-to-transmittance ratio across a broad frequency range. As discussed earlier, a broadband beamsplitter with a tunable splitting ratio may be realized using a single-layer metamaterial that exhibits perfect broadband reflection for *θ* = 0° and perfect broadband transmission for *θ* = 45°. To test this, we designed the structure shown in Figure 2(c) as a constituent element. The dipole resonator acts as a mirror at around the resonance frequency for *θ* = 0° as shown in Figure 3(a). Therefore, it is easy to realize broadband high reflection for *θ* = 0° using the structure shown in Figure 2(c) as long as this two-layer structure is designed so that the Fabry–Perot resonance does not occur [57]. Although the structure is visibly two-layer, we hypothesize that it behaves effectively as a single-layer structure, provided its thickness remains much smaller than the wavelength of the incident electromagnetic wave.

**Figure 4:**
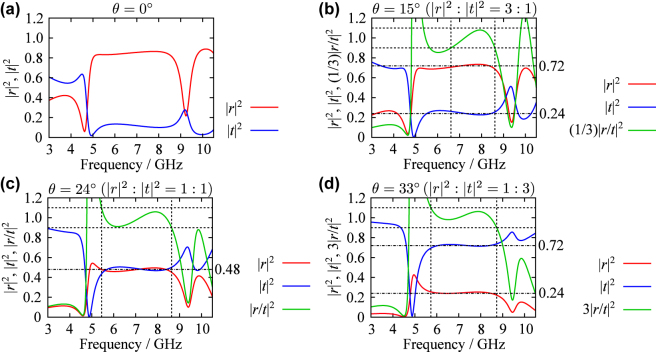
Numerically calculated reflection and transmission spectra of the two-layer metamaterial for (a) *θ* = 0°, (b) 15°, (c) 24°, and (d) 33°. The horizontal dashed lines indicate the region where the deviation of the ratio of |*r*|^2^ to |*t*|^2^ from the intended value (shown above each graph) is less than 10 %, and the vertical dashed lines indicate the corresponding frequency range.

We numerically analyzed the reflection and transmission spectra of the metamaterial composed of this structure for various values of *θ*. Figure 4(a)–(d) shows the computed spectra for *θ* = 0°, 15°, 24°, and 33°. For *θ* = 0°, the reflectance |*r*|^2^ exceeds 0.8 across the frequency range from 5.01 GHz to 8.64 GHz. Although perfect reflection is not achieved, the reflectance remains high throughout this range. As *θ* increases, |*r*| decreases and |*t*| increases, consistent with the behavior observed in the single-layer metamaterial. The reflectance-to-transmittance ratios for *θ* = 15°, 24°, and 33° are approximately 3 ± 0.3 (6.61 GHz–8.61 GHz), 1 ± 0.1 (5.45 GHz–8.64 GHz), and 1/3 ± 1/30 (5.73 GHz–8.73 GHz), respectively. In these frequency ranges, the sum |*r*|^2^ + |*t*|^2^ is approximately 0.96, indicating low absorbance (∼0.04) despite the relatively high dielectric losses of the substrate and superstrate. These results confirm that a broadband beamsplitter with a splitting ratio controlled by *θ* can be realized using the conjectured method. Although the theory assumes single-layer metamaterials, the numerical results demonstrate that the theoretical predictions are approximately valid for two-layer metamaterials with subwavelength thickness.

## 4 Conclusions

We have proposed and numerically validated using COMSOL Multiphysics that the reflectance-to-transmittance ratio of metamaterials with subwavelength thickness can be controlled by varying the spatial arrangement of the constituent elements, without altering their individual structures. The developed theory demonstrates that this ratio at the resonance frequency can be arbitrarily tuned by adjusting the parameter *θ* in single-layer metamaterials. Building on this result, we conjectured a method for realizing beamsplitters with a frequency-independent splitting ratio governed by *θ*, and numerically confirmed that a broadband variable beamsplitter can be realized using a two-layer metamaterial with subwavelength thickness.

We used a metamaterial composed of the two-layer structure to realize a broadband variable beamsplitter because it was easy to design a metamaterial that exhibits broadband high reflection for *θ* = 0° based on the two-layer structure. However, according to the proposed theory, the variable beamsplitter can be realized with the use of single-layer structures. For future work, it is essential to design a broadband variable beamsplitter composed of a single-layer structure that exhibits broadband high reflection for *θ* = 0°. In addition, it is necessary to develop a method for fabricating single-layer metamaterials with variably controllable *θ*. The value of *θ* can only be varied using mechanical methods; thus, the proposed variable beamsplitter could be fabricated in the frequency region up to, at most, terahertz frequencies.

Although this study was limited to configurations where diffraction does not occur, the concept may be extended to realize multiport variable beamsplitters by incorporating diffraction effects. The radiation from a metamaterial depends on the relationship between the radiation pattern and the phase distribution of the induced dipoles in the meta-atoms, which holds regardless of whether diffraction occurs or not. This implies that the port number of the variable beamsplitter could be increased by increasing the period of the metamaterial so that diffraction occurs. These findings contribute to the advancement of electromagnetic wave applications in areas such as information processing, precision measurement, and imaging.
